# *Geosesarmamirum*, a new species of semi-terrestrial sesarmid crab (Crustacea, Decapoda, Brachyura) from central Taiwan

**DOI:** 10.3897/zookeys.858.35198

**Published:** 2019-07-01

**Authors:** Jhy-Yun Shy, Peter K.L. Ng

**Affiliations:** 1 Department of Aquaculture, National Penghu University of Science and Technology, Penghu 880, Taiwan National Penghu University of Science and Technology Taipei Taiwan; 2 Lee Kong Chian Natural History Museum, National University of Singapore, 2 Conservatory Drive, Singapore 117377, Republic of Singapore National University of Singapore Singapore Singapore

**Keywords:** Direct development, East Asia, freshwater, large eggs, Sesarmidae, taxonomy

## Abstract

A new species of semi-terrestrial sesarmid crab of the genus *Geosesarma* De Man, 1892, is described from central Taiwan. *Geosesarmamirum***sp. nov.** is distinct in possessing a strong transverse crest on the inner surface of the male chela and a diagnostic male first gonopod which is relatively long and stout, with the distal chitinous part broad and spatuliform. Like most *Geosesarma* species, *G.mirum***sp. nov.** has large eggs and direct development, contrasting with the only other species known from Taiwan, *G.hednon* Ng, Liu and Schubart, 2004, which has small eggs and planktotrophic larvae.

## Introduction

*Geosesarma* De Man, 1892, is a large genus of semi-terrestrial and terrestrial crabs occurring in many freshwater habitats in Southeast Asia, Andamans, and western Pacific. At the moment, 64 species have been recorded, with the majority from Indonesia (Ng et al. 2008; Ng 2015, 2017; Ng et al. 2015; Manuel-Santos et al. 2016; Ng and Grinang 2018; Ng and Wowor 2019; Ng and Ng 2019). Thus far, the most northerly record of the genus is Taiwan and Luzon in the Philippines, with two known species: *G.vicentense* (Rathbun, 1914) and *G.hednon* Ng, Liu & Schubart, 2004 (Ng et al. 2004; Ng and Lemaitre 2017). Both species, however, are atypical members of *Geosesarma* as they have small eggs and planktotrophic larvae (see Ng et al. 2004; Ng and Lemaitre 2017). Most species of *Geosesarma* (when egg sizes are known) (over 90%) have large eggs and direct development.

In late 2018, the authors became aware of a semi-terrestrial freshwater sesarmid living in the lowlands of Chiayi County in central-western Taiwan with large eggs. Specimens were eventually obtained and studies showed that they belonged to a new species of *Geosesarma*. This is the first record of a large-egged *Geosesarma* from such a high latitude. The description of the new species and comparisons with congeners form the basis of the present paper.

## Materials and methods

The terminology used here follows Ng (1988) and Davie et al. (2015). Measurements provided are the carapace width and length, respectively. The abbreviations G1 and G2 are used for the male first and second gonopods, respectively. Specimens examined are deposited in the zoology collections of the National Taiwan Ocean University (**NTOU**), Keelung, Taiwan; and the Zoological Reference Collection (**ZRC**) of the Lee Kong Chian Natural History Museum, National University of Singapore. Specimens with eggs or young crabs were not collected and released after study.

## Systematic accounts

### Family Sesarmidae Dana, 1851

#### Genus Geosesarma

Taxon classificationAnimaliaDecapodaSesarmidae

De Man, 1892

##### Type species.

Sesarma (Geosesarma) nodulifera De Man, 1892, subsequent designation by Serène and Soh (1970).

#### Geosesarma mirum sp. nov.

Taxon classificationAnimaliaDecapodaSesarmidae

http://zoobank.org/60FAAFB1-8BC8-416C-AF80-295A6EEA45D0

**Figure 1.**
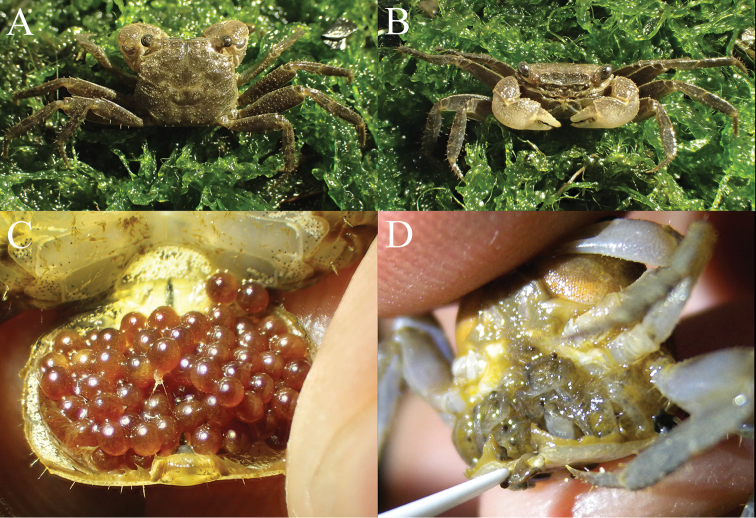
*Geosesarmamirum* sp. nov. **A, B** male (9.9 × 8.6 mm) (NTOU F10396) **C** ovigerous female with eggs (specimen not collected) **D** brooding female with young crabs (specimen not collected). Photographs **A–C** J-Y Shy, **D** Peter Wang.

**Figure 2.**
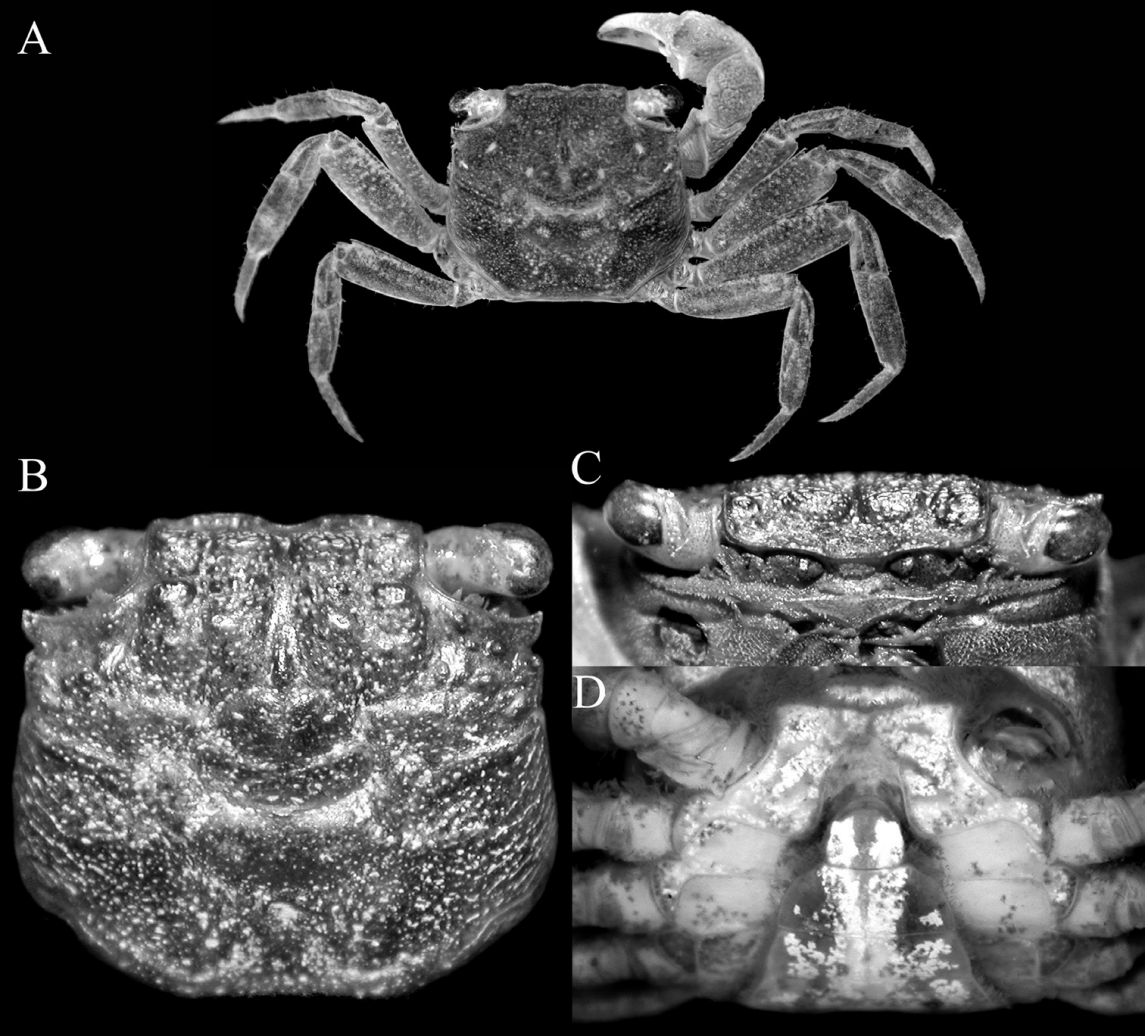
*Geosesarmamirum* sp. nov. holotype male (11.9 × 10.8 mm) (NTOU F10395), Taiwan **A** overall dorsal view **B** dorsal view of carapace **C** frontal view of cephalothorax **D** anterior thoracic sternum and pleon.

**Figure 3.**
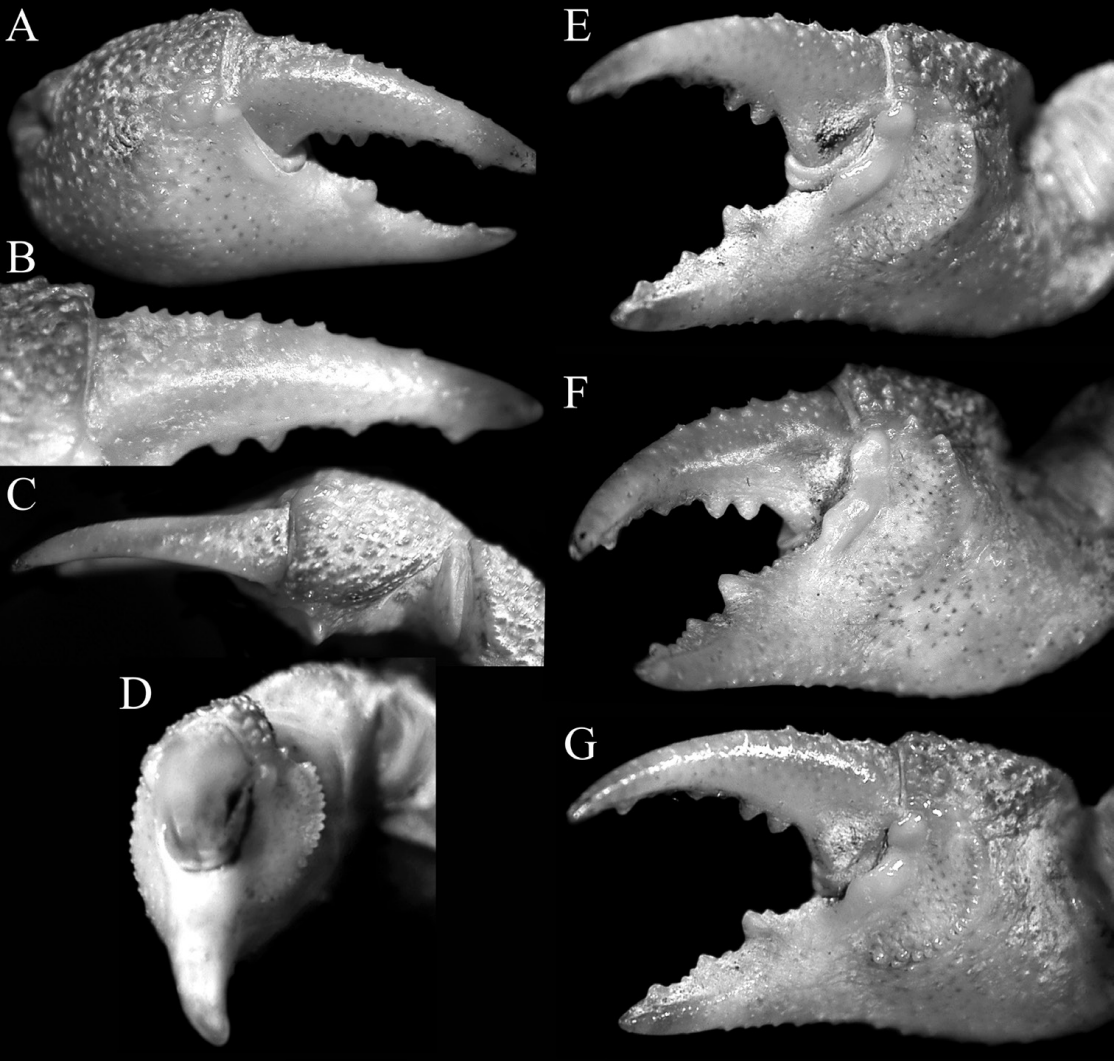
*Geosesarmamirum* sp. nov. holotype male (11.9 × 10.8 mm) (NTOU F10395), Taiwan. Right chela **A** outer view **B** outer view of dactylus **C** dorsolateral view **D** frontolateral view showing crest on inner surface **E–G** different views of inner surface.

**Figure 4.**
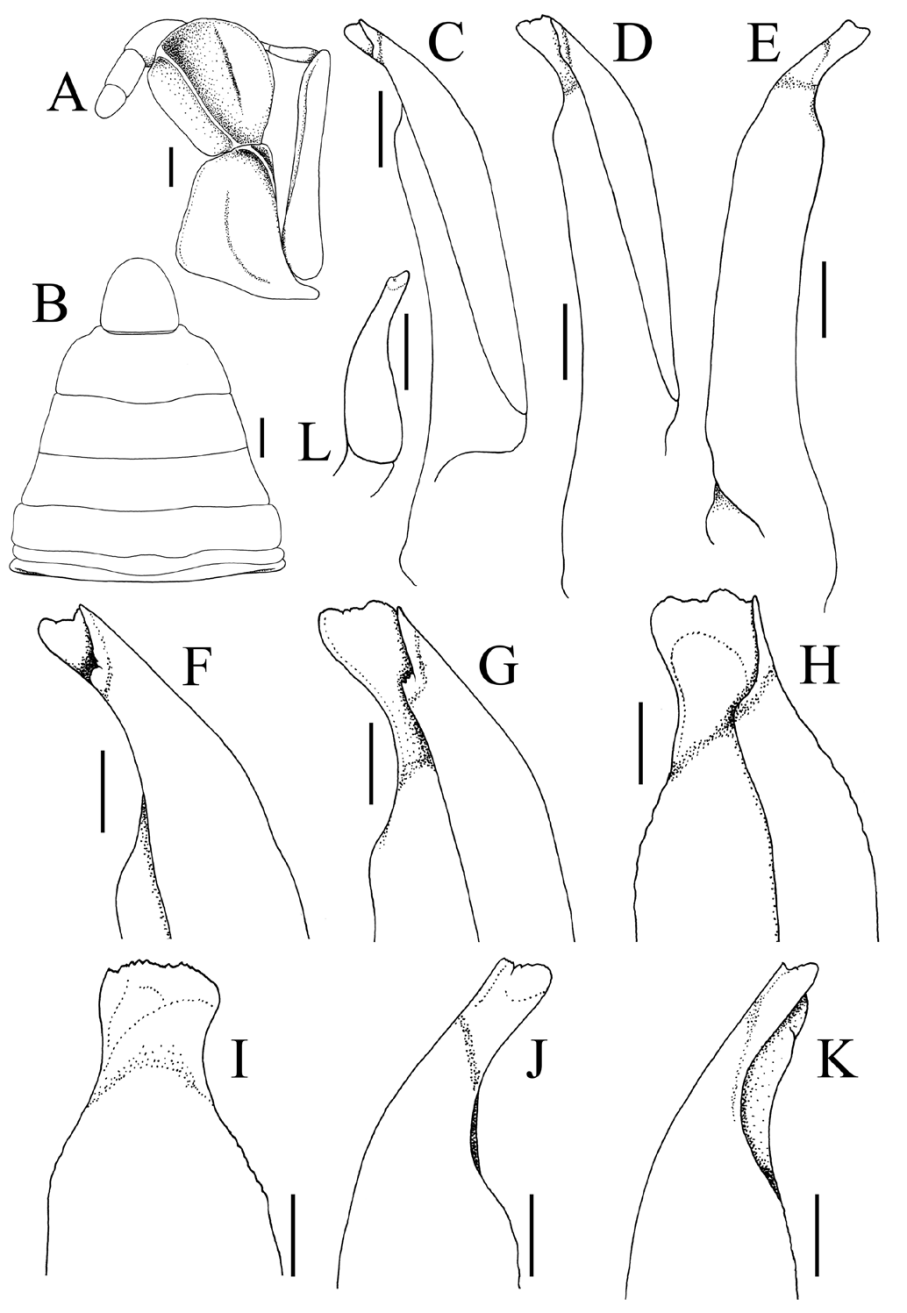
*Geosesarmamirum* sp. nov. holotype male (11.9 × 10.8 mm) (NTOU F10395), Taiwan **A** left third maxilliped **B** pleon **C, D** different views of ventral surface of left G1 **E** dorsal surface of left G1 **F–H** different views of ventral surface of distal part of eft G1 **I** mesial view of distal part of left G1 **J, K** different views of dorsal surface of distal part of left G1 **L** left G2. Scale bars: 0.5 mm (**A, C, D, L**); 1.0 mm (**B**); 0.2 mm (**F–K**).

**Figure 5.**
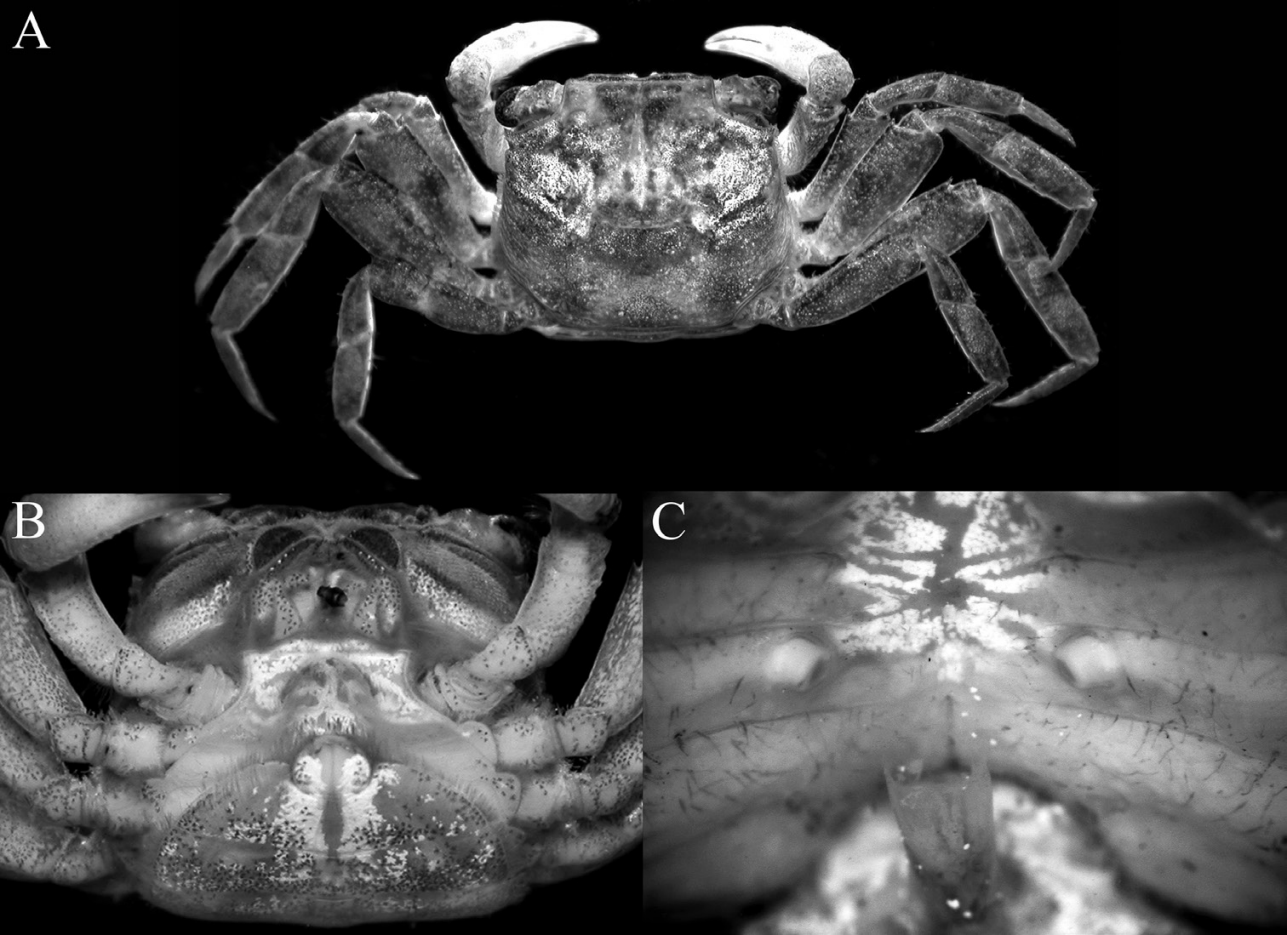
*Geosesarmamirum* sp. nov. paratype female (10.6 x 9.2 mm) (ZRC 2019.0513), Taiwan **A** overall dorsal view **B** anterior thoracic sternum and pleon **C** sternopleonal cavity and vulvae.

Figures 1 , 2 , 3 , 4 , 5

##### Material examined.

Holotype: TAIWAN - male (11.9 x 10.8 mm); Chiayi County, Jhongpu, Lunziding Canal; 23.44914N 120.48227E; 28 Jan. 2019, leg. J.-Y. Shy; under rocks near stream; NTOU F10395. Paratypes: TAIWAN - 3 males (11.5 x 10.3 mm, 10.1 × 8.9 mm, 9.9 × 8.7 mm), 5 females (12.2 × 10.1 mm, 10.6 × 9.2 mm, 9.9 × 8.9 mm, 9.1 × 7.7 mm, 7.9 × 7.2 mm), 1 juvenile female (6.6 × 5.7 mm); same data as holotype; ZRC 2019.0513. 2 males (9.9 × 8.6 mm, 8.4 × 7.3 mm); Chiayi County, Jhongpu, Chilan River; 23.43744N 120.48917E ; 21 Febr. 2019, leg. J.-Y. Shy; NTOU F10396. 1 male (11.5 x 9.8 mm); Chiayi County, Jhongpu, branch of Chilan River; ca. 23.43744N 120.48917E; 22 March 2019; leg. J.-Y. Shy; NTOU F10397. 7 males (7.8 x 6.5 mm – 12.2 x 11.2 mm), 5 females (9.3 x 7.8 mm – 11.4 x 10.0 mm), same locality as holotype; 23 March 2019; leg. J.-Y. Shy & H.-T. Lai; ZRC 2019.0514.

##### Diagnosis.

Carapace quadrate, slightly wider than long or subequal, adult width to length ratio 1.10–1.21, lateral margins gently sinuous, gently diverging posteriorly (Fig. 2A, B); dorsal surface with regions visible, anterior regions with small rounded granules on gastric regions, branchial regions with numerous striae (Fig. 2A, B); front distinctly deflexed, frontal lobes broad, with subtruncate margins in dorsal view; postfrontal, postorbital cristae prominent, rugose (Fig. 2A–C); external orbital tooth triangular to subtruncate, directed obliquely laterally, outer margin convex, shorter than inner margin, tip reaching lateral margin; second lateral tooth low, rounded, separated from external orbital tooth by deep notch (Fig. 2A, B). Merus of third maxilliped subovate, subequal to ischium; exopod slender, reaching to just before edge of merus, with long flagellum (Fig. 4A). Merus of cheliped with low ventral lobe with serrated margin, upper lobe relatively lower. Outer surface of palm of adult male covered with small rounded granules and striae; inner surface granulated, with distinct, high transverse granulated ridge; dorsal margin of dactylus with 11 or 12 low, non-chitinous tubercles on proximal two-thirds (Fig. 3). Ambulatory legs with relatively stout, short merus, with sharp subdistal spine on dorsal margin, surfaces rugose (Fig. 1A). Part of male thoracic sternite 8 exposed when pleon closed. Male pleonal locking mechanism formed by expanded posterior edge of thoracic sternite 4. Male pleon triangular; somite 6 wide, with convex lateral margins; telson triangular, not recessed into distal margin of somite 6, margins convex (Figs 2D, 4B). G1 relatively long, stout, gently curved outwards; outer margin of subdistal part of subterminal segment with subangular shelf-like structure (Fig. 4C, D), distal chitinous part broad, tip spatuliform, margin uneven (Fig. 4C–K). G2 short, ca. a third length of G1 (Fig. 4L).

##### Etymology.

The name is derived from the Latin for “surprise”, alluding to the unexpected discovery of this species in Taiwan.

##### Remarks.

*Geosesarmamirum* sp. nov. belongs to the group of species which have large eggs (ca. 1.0 mm or greater in diameter, measured in situ), the carapace is trapezoidal to subquadrate, the ambulatory meri are relatively short and stout, the exopod of the third maxilliped has a long flagellum, the inner surface of the male chela has a strong granulated transverse ridge and the G1 is relatively stout with the distal chitinised part spatuliform. The species in this group are: *G.amphinome* (De Man, 1899) [western Borneo], *G.peraccae* (Nobili, 1903) [Singapore and Peninsular Malaysia], *G.penangense* (Tweedie, 1940) [Penang, Peninsular Malaysia], *G.sarawakense* (Serène, 1968) [Sarawak, Borneo] and *G.pylaemenes* Ng, 2015 [western Borneo]. *Geosesarmamirum* can be distinguished from these species mainly by the distinctive form of its G1. Compared to *G.amphinome*, the distal chitinised part of the G1 of *G.mirum* is distinctly shorter and the tip is not bilobed (cf. Ng 2015: fig. 1A, B, 2A, G–K). Compared to *G.peraccae*, the G1 of *G.mirum* is stouter overall with the chitinous part proportionately much shorter (cf. Ng 1988: fig. 56A, D–F; Ng 2015: fig. 5A, B). The G1 of *G.mirum* differs from that of *G.penangense* in having the distal part gently curved rather than strongly bent, with the ambulatory leg merus proportionately stouter (cf. Ng 1988: fig. 58A, D, E). When compared to *G.sarawakense*, the carapace of *G.mirum* is distinctly more granulated and rugose, with the G1 proportionately stouter and shorter (Ng 2015: figs 6A, B, 7D–F). In contrast to *G.pylaemenes*, the external orbital tooth of *G.mirum* is more acute and the G1 is relatively stouter (cf. Ng 2015: fig. 3A, B, 4D–G).

In Taiwan and other parts of the Indo-West Pacific, *Geosesarmamirum* can be confused with species of *Scandarma* Schubart, Liu & Cuesta, 2003 (type species *Scandarmalintou* Schubart, Liu & Cuesta, 2003), and *Pseudosesarma* Serène & Soh, 1970 (type species *Sesarmaedwardsii* De Man, 1887) because in these genera, the male chelipeds do not have pectinated ridges on their chelae and there are no stridulatory granules on the dorsal margin of the dactylus. *Geosesarmamirum* can be easily distinguished from species of *Scandarma* as the outer surface of the male chela does not have a distinct swelling and the G1 is very short and stout (see Schubart et al. 2003; Naruse and Ng 2007; Ng 2013; Naruse and Ng 2019). From *Pseudosesarma*, many members of which live in freshwaters, *G.mirum* can be distinguished by the G1 morphology, with those of *Pseudosesarma* species short, very stout with the median or distal parts prominently dilated and with sharp chitinised “beaks” (see Ng and Schubart 2017).

**Figure 6.**
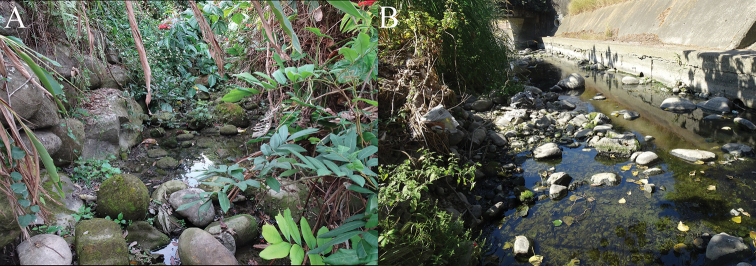
Habitat of *Geosesarmamirum* sp. nov. **A** Chilan River, Jhongpu, Chiayi County, Taiwan, 23.43744N, 120.48917E**B** Lunziding Canal, Jhongpu, Chiayi County, Taiwan, 23.44914N, 120.48227E.

##### Ecology.

*Geosesarmamirum* sp. nov. has a semi-terrestrial habit and has been found in small streams in lowlands, with the crabs digging burrows under stones near the edge of the water. The first author investigated 28 sites in and around the type locality of species, in the Ba-Jhang River region (total area of ca. 200 km^2^). Most of the sites examined were badly polluted, cemented, built over or no longer had permanent water. There were some sites with clean water but these were very close to the hills (altitude higher than ca. 100 m) but these sites only had the primary freshwater crab, *Geothelphusaolea* Shy, Ng & Yu, 1994 (Potamidae). Of the 28 sites surveyed, *Geosesarmamirum* was only found in six sites (in an area of ca. 4 km^2^). These six sites were from different branches of the river and the crabs were relatively abundant in each of these locations (sometimes more than 50 individuals/m^2^). From the surveys done, the species seems to have a relatively localised distribution, with some of the sites where they were found only a few dozen square metres in area, and the crabs absent from sites further upstream or downstream. This may not be the natural situation as the sites where the crabs were absent were invariably badly polluted or extensively concreted.

The development of *G.mirum* is direct (i.e., abbreviated), with the eggs measuring ca. 1.0 mm in diameter (specimen not preserved) (Fig. 1C). The egg sizes of *Geosesarma* species average between 1.2 and 1.8 mm (Ng 2017; Ng and Wowor 2019). Despite the slightly smaller egg size, we have observed females of *G.mirum* brooding young crabs under the pleon (Fig. 1D) and it is clear that the development is completely abbreviated (see Ng and Tan 1995). This contrasts with catadromous grapsoid species like *Eriocheirjaponica* (De Haan, 1835) (Varunidae) which have much smaller eggs (ca. 0.34 mm; cf. Lai et al. 1986). Their eggs, however, are still smaller than those of primary freshwater species like *Nanhaipotamonformosanum* (Parisi, 1916) (Potamidae) which have egg diameters of up to 4.0 mm (cf. Lai et al. 2012).

## Supplementary Material

XML Treatment for Geosesarma

XML Treatment for Geosesarma mirum
